# Supplementary material for: Analysis of mutations in precision oncology using the automated, accurate, and user-friendly web tool PredictONCO
Source: Comput Struct Biotechnol J. 2024 Nov 14;24:734–8. doi: 10.1016/j.csbj.2024.11.026 (PMC11647622; doi:10.1016/j.csbj.2024.11.026)
Supplement: Supplementary file 1 — Supplementary material [file mmc1.docx]

**Analysis of Mutations in Precision Oncology using The Automated, Accurate, and User-Friendly Web Tool PredictONCO**

Rayyan Tariq Khan^1,2,#^, Petra Pokorna^4,6#^, Jan Stourac^1,2^, Simeon Borko^1,2,3^, Adam Dobias^1^, Joan Planas-Iglesias^1,2^, Stanislav Mazurenko^1,2^, Ihor Arefiev^1^, Gaspar Pinto^1,2^, Veronika Szotkowska^1^, Jaroslav Sterba^5^, Jiri Damborsky^1,2^, Ondrej Slaby^4,6*^, David Bednar^1,2,*^

^1^Loschmidt Laboratories, Department of Experimental Biology and RECETOX, Faculty of Science, Masaryk University, Brno, Czech Republic; ^2^International Clinical Research Center, St. Anne’s University Hospital Brno, Brno, Czech Republic, ^3^IT4Innovations Centre of Excellence, Faculty of Information Technology, Brno University of Technology, Brno, Czech Republic, ^4^Department of Biology, Faculty of Medicine and Central European Institute of Technology, Masaryk University, Brno, Czech Republic, ^5^Department of Paediatric Oncology, University Hospital Brno and Faculty of Medicine, Masaryk University, Brno, Czech Republic; ^6^ Department of Biology, Faculty of Medicine, Masaryk University, Brno, Czech Republic; # joint first authors; * authors for correspondence: Ondrej Slaby - [on.slaby@gmail.com](mailto:on.slaby@gmail.com); David Bednar – [davidbednar1208@gmail.com](mailto:davidbednar1208@gmail.com)

**Supplementary Materials.**

***New proteins, datapoints, and predictors training.***

Our previous predictor was trained on 44 proteins, on a dataset that contained 509 oncogenic and 564 benign data points. All these data points contained sequential data (SEQ). For approximately half of the data (377 oncogenic and 76 benign data points) structural information was available, conforming the structural (STR) dataset. In order to work with an appropriate training/validation/testing data split, 20% of the data was kept aside for testing, chosen randomly but grouped by positions to ensure that no specific position in a protein from the test set appears in the training set. Three different kinds of predictors were trained this way: a support vector machine (SVM), a decision tree (DT), and a XGBoost classifier (XGB), taken as they are implemented in the scikit-learn 1.2.0 and xgboost 1.7.3 libraries for Python 3.8.15. The training consisted of testing a set of hyperparameters on a 5-fold cross-validation procedure optimising the area under the receiver operating characteristic (ROC) curve (AUROCC) as the metric. Our best predictor, XGB, achieved 0.93 +/- 0.02 AUC and 0.93 +/- 0.02 area under the precision-recall (PR) curve (AUPRC). When considering only the dataset with structural information, the performance was 0.96 +/- 0.02 AUROCC, and 0.99 +/- 0.01 AUPRC [1,2].

On the current release, we increased the number of proteins used for training to 52, incorporating 8 new proteins (**Supplementary Table 1**). All these new proteins are available now in the web server to be enquired about the effect of new mutations on them. We obtained annotations for these proteins generating 269 new data points, of which 189 contained structural information (**Supplementary Table 2**). We relied on the previous hyperparameter optimisation [1,2] to re-train our predictors (DT, SVM, and XGB) with the new data points exploiting separately sequential and structural information (using the SEQ and STR datasets, respectively), and we performed the training using the same protocol as used before [1,2] (and explained above). XGB remained the best predictor for the structural data (**Supplementary Figure 1**), achieving 0.94 +/- 0.01 AUROCC, and 0.94 +/- 0.01 AUPRC for the whole dataset and 0.97 +/- 0.01 AUROCC, and 0.99 +/- 0.01 AUPRC when considering only data points with structure information. SVM was slightly better for the SEQ dataset (**Supplementary Figure 2**), but the improvement was within the error margin when compared to XGB.

**Supplementary Table 1**. New proteins in PredictONCO

| **Protein Name** | **UniProt Accession Code** | **Effect in Tumor** | **Protein Description** |
| --- | --- | --- | --- |
| BRAF | P15056 | Proto-oncogenic | Serine/threonine-protein kinase B-raf |
| CALR | P27797 | Suppressor | Calreticulin |
| CTNNB1 | P35222 | Proto-oncogenic | Catenin beta-1 |
| H3F3A | P84243 | Proto-oncogenic | Histone H3.3 |
| IDH2 | P48735 | Proto-oncogenic | Isocitrate dehydrogenase |
| MPL | P40238 | Proto-oncogenic | Thrombopoietin receptor |
| POLE | Q07864 | Proto-oncogenic | DNA polymerase epsilon catalytic subunit A |
| TERT | O14746 | Proto-oncogenic | Telomerase reverse transcriptase |

**Supplementary Table 2**. Distribution of new datapoints in PredictONCO

| **Mutation Type** | **Dataset** | |
| --- | --- | --- |
|  | **Sequential (SEQ)** | **Structural (STR)** |
| **Benign** | 141 | 104 |
| **Oncogenic** | 128 | 85 |
| **Total** | 269 | 189 |

**Supplementary Figure 1**.


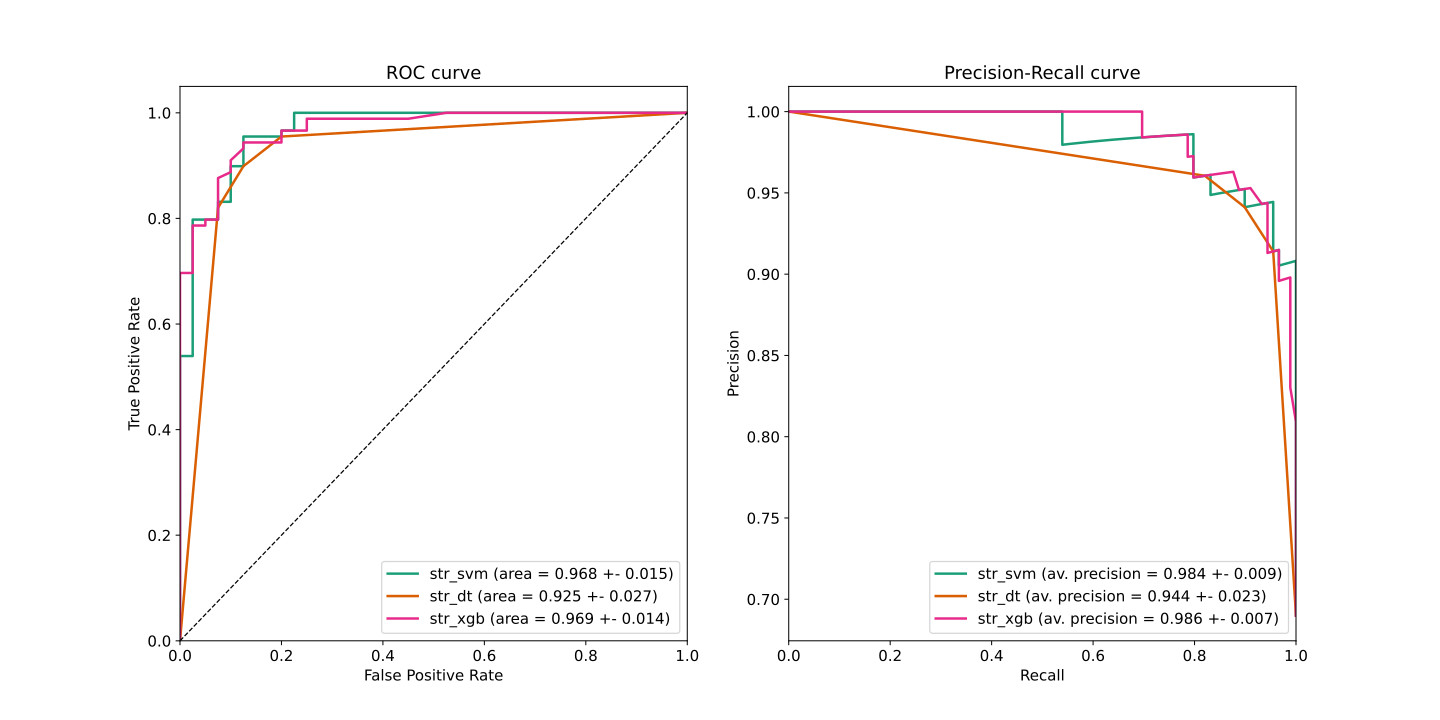


**ROC and Precision-Recall curves for the predictors trained with structural data (STR dataset).** DT (orange), SVM (green) and XGB (magenta) predictors performance on the structural dataset in the form of ROC (right) and Precision-Recall (left) curves. XGB remains the best predictor for structural data.

**Supplementary Figure 2**.


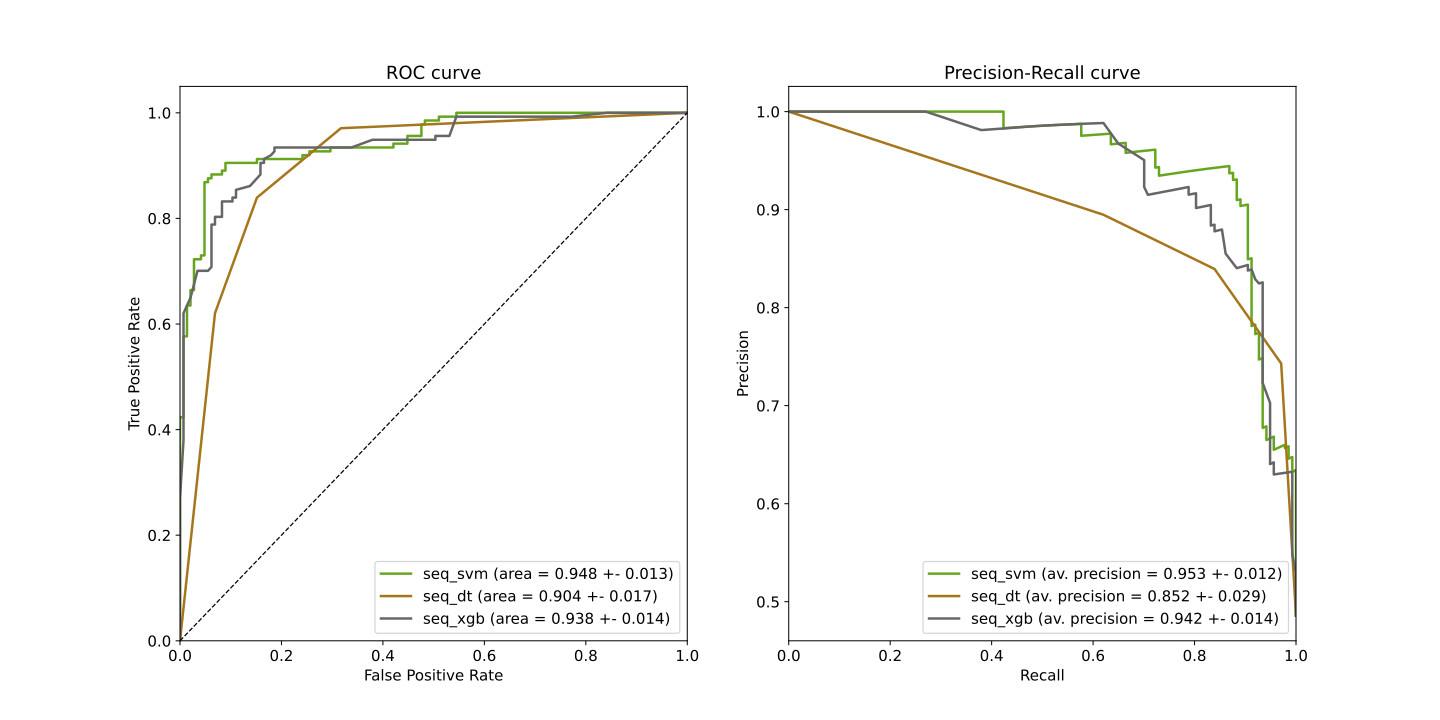


**ROC and Precision-Recall curves for the predictors trained with sequential data (SEQ dataset).** DT (brown), SVM (green) and XGB (gray) predictors performance on the structural dataset in the form of ROC (right) and Precision-Recall (left) curves. SVM outperforms XGB, but the improvement remains within the error margin.

**References**

[1] Stourac, J., Borko, S., Khan, R.T., Pokorna, P., Dobias A., Planas-Iglesias, J., Mazurenko, S., Pinto, G., Szotkowska, V., Sterba, J., Slaby, O., Damborsky, J., Bednar, D. (2023). PredictONCO: a web tool supporting decision-making in precision oncology by extending the bioinformatics predictions with advanced computing and machine learning. *Briefings in Bioinformatics*. 25:bbad441.

[2] Khan, R.T., Pokorna, P., Stourac, J., Borko, S., Arefiev I., Planas-Iglesias, J., Dobias, A., Pinto, G., Szotkowska, V., Sterba, J., Slaby, O., Damborsky, J., Mazurenko, S., Bednar, D. (2024). A computational workflow for analysis of missense mutations in precision oncology. *J Cheminform.* 16:86.
